# Potential role of oxidative stress-induced apoptosis in mediating chromosomal rearrangements in nasopharyngeal carcinoma

**DOI:** 10.1186/s13578-016-0103-9

**Published:** 2016-05-25

**Authors:** Sang-Nee Tan, Sai-Peng Sim, Alan S. B. Khoo

**Affiliations:** Faculty of Medicine and Health Sciences, Department of Paraclinical Sciences, Universiti Malaysia Sarawak, Sarawak, Malaysia; Molecular Pathology Unit, Cancer Research Centre, Institute for Medical Research, Kuala Lumpur, Malaysia

**Keywords:** *NPC*, *Oxidative stress*, *H*_*2*_*O*_*2*_, *Apoptosis*, *AF9 gene*, *CAD*

## Abstract

**Background:**

Genetic aberrations have been identified in nasopharyngeal carcinoma (NPC), however, the underlying mechanism remains elusive. There are increasing evidences that the apoptotic nuclease caspase-activated deoxyribonuclease (CAD) is one of the players leading to translocation in leukemia. Oxidative stress, which has been strongly implicated in carcinogenesis, is a potent apoptotic inducer. Most of the NPC etiological factors are known to induce oxidative stress. Although apoptosis is a cell death process, cells possess the potential to survive apoptosis upon DNA repair. Eventually, the surviving cells may carry rearranged chromosomes. We hypothesized that oxidative stress-induced apoptosis may cause chromosomal breaks mediated by CAD. Upon erroneous DNA repair, cells that survive apoptosis may harbor chromosomal rearrangements contributing to NPC pathogenesis. This study focused on the *AF9* gene at 9p22, a common deletion region in NPC. We aimed to propose a possible model for molecular mechanism underlying the chromosomal rearrangements in NPC.

**Results:**

In the present study, we showed that hydrogen peroxide (H_2_O_2_) induced apoptosis in NPC (HK1) and normal nasopharyngeal epithelial (NP69) cells, as evaluated by flow cytometric analyses. Activity of caspases 3/7 was detected in H_2_O_2_-treated cells. This activity was inhibited by caspase inhibitor (CI). By nested inverse polymerase chain reaction (IPCR), we demonstrated that oxidative stress-induced apoptosis in HK1 and NP69 cells resulted in cleavages within the breakpoint cluster region (BCR) of the *AF9* gene. The gene cleavage frequency detected in the H_2_O_2_-treated cells was found to be significantly higher than untreated control. We further found that treatment with CI, which indirectly inhibits CAD, significantly reduced the chromosomal breaks in H_2_O_2_-cotreated cells. Intriguingly, a few breakpoints were mapped within the *AF9* region that was previously reported to translocate with the mixed lineage leukemia (*MLL*) gene in acute lymphoblastic leukemia (ALL) patient.

**Conclusions:**

In conclusion, our findings suggested that oxidative stress-induced apoptosis could be one of the mechanisms underlying the chromosomal rearrangements in NPC. CAD may play an important role in chromosomal cleavages mediated by oxidative stress-induced apoptosis. A potential model for oxidative stress-induced apoptosis mediating chromosomal rearrangements in NPC is proposed.

## Background

Nasopharyngeal carcinoma (NPC) is a solid malignancy which demonstrates a unique ethnic and geographic distribution. In most parts of the world, its incidence rates are below one per 100,000 persons per year. However, it has a notable exception in Southern China and South-East Asia [1, 2]. The highest rates were reported among Southern Chinese living in central Guangdong province, the annual incidence rates for males and females are 23.3/100,000 and 8.9/100,000, respectively [3]. More recently, high incidence of NPC (23.1/100,000/year) has also been observed among the native Bidayuh people in Sarawak, one of the two Malaysian states on the Borneo Island [4].

The striking geographic and ethnic distribution of NPC suggests that pathogenesis of NPC is a multi-step process involving multiple factors. It has been recognized that NPC is strongly associated with Epstein-Barr Virus (EBV) infection [5], dietary factors [6], environmental exposure [7], epigenetic modifications [8] and genetic alterations [9]. Various approaches have been made to identify common chromosomal anomalies in NPC, and yet the molecular mechanism(s) underlying the chromosomal rearrangements remains enigmatic. Well illustration of the molecular basis of NPC pathogenesis is essential in identifying potential therapeutic targets, so that a more effective and specific therapy may be developed for NPC.

Nowadays, there are increasing evidences that the apoptotic nuclease participates in the initial event of chromosome translocation in leukemia, that is the chromosome breakage [10, 11]. Thus, it is important to investigate the role of apoptosis in NPC chromosomal rearrangements. Apoptosis can be triggered by oxidative stress [12] which is caused by excessive production of reactive oxygen species (ROS) such as hydrogen peroxide H_2_O_2_, hydroxyl radical OH**·**, superoxide O^2−^**·** and peroxyl RO**·**. ROS are constantly generated as a consequence of aerobic respiration [13]. ROS are also involved in a variety of normal cellular processes such as wound healing and anti-bacterial defense. However, excessive formation of ROS has been implicated in various diseases such as cancer, diabetes and neurodegenerative disorder (reviewed in [14]). Oxidative stress stemming from chronic inflammation is well known to be involved in carcinogenesis [15]. Chronic inflammation caused by EBV infection is associated with radical-mediated DNA damage. Oxidative DNA lesions and nitrative DNA lesions were detected in cancer cells and inflammatory cells in stroma of NPC patients [16]. It has been demonstrated that the EBV nuclear antigen (EBNA)-1 induced chromosomal aberrations, DNA double-strand breaks (DSBs) and engagement of the DNA damage response (DDR) which were associated with formation of ROS and were reversed by antioxidants [17]. Long-term exposures to nitrosamine, wood dust, formaldehyde and cigarette smoke have all been thought to be etiological factors of NPC [7, 18–20]. Interestingly, all these etiological factors have been found to induce oxidative stress which may in turn lead to genomic instability [21–24].

Although apoptosis is a cell death process, cells have the potential and tendency to recover upon DNA repair [25]. In human cells, chromosomal DSBs are primarily repaired by non-homologous end joining (NHEJ) DNA repair system. This pathway joins two ends of a DSB via a process mainly independent of terminal DNA sequence homology. Instead, a few nucleotides of homology called terminal microhomology (typically one to four nucleotides) are usually utilized by NHEJ enzymes [26, 27]. Therefore, this pathway is prone to cause erroneous repair of DSBs which may result in chromosomal deletions, insertions or translocations as demonstrated by Weinstock and colleagues [28].

During apoptotic process, members of caspase family of proteases are being activated and consequently cause the fragmentation of genomic DNA via a cascade of proteolytic events. A 40 kD caspase-activated deoxyribonuclease (CAD) which can be activated by caspase-3 exists naturally as a heterodimer with a 45 kD chaperone, inhibitor of CAD (ICAD) in non-apoptotic cells. When caspases particularly caspase-3 are activated by apoptotic stimuli, the ICAD which possesses two caspase-3 cleavage sites will be cleaved by caspase-3 [29, 30]. After the cleaved ICAD is being released from CAD, it allows CAD to oligomerize and form a large functional complex which cleaves DNA by generating DSBs. Histone H1 interacts directly with CAD, confers DNA binding ability to CAD and stimulates its nuclease activity [31].

In this study, we report that oxidative stress induced apoptosis in normal nasopharyngeal epithelial and NPC cells. We demonstrate that oxidative stress-induced apoptosis triggered caspase activity that was inhibited by caspase inhibitor (CI). We also present evidence that oxidative stress-induced apoptosis resulted in chromosomal breaks in the *AF9* gene. This gene was targeted because it is located at 9p22, a common deletion site in NPC [32]. Intriguingly, a few breakpoints were mapped within the *AF9* region that was previously reported to be involved in t(9;11)(p22;q23) in acute lymphoblastic leukemia (ALL) patient. We further demonstrate that CI significantly reduced oxidative stress-induced chromosomal breaks, suggesting a role of CAD in mediating the chromosomal breaks during oxidative stress. Finally, we propose a potential model for oxidative stress-induced apoptosis in mediating chromosomal rearrangements in NPC.

## Results

### Hydrogen peroxide (H_2_O_2_) induces phosphatidylserine (PS) externalization in HK1 and NP69 cells

**Fig. 1:**
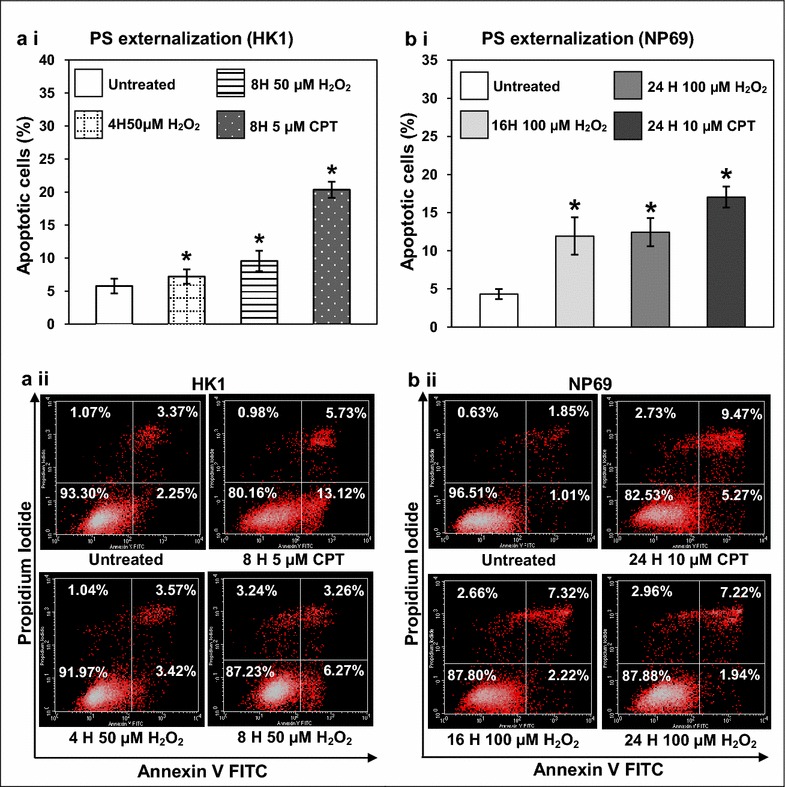
H_2_O_2_ induces PS externalization in HK1 and NP69 cells. HK1 cells were either left untreated or treated with 50 µM of H_2_O_2_ for 4 and 8 h, whereas NP69 cells were either left untreated or treated with 100 µM of H_2_O_2_ for 16 and 24 h. The cells were then subjected to flow cytometric analysis of PS externalization as described in “Methods” section. Cells treated with CPT were included as a positive control. Percentages of apoptotic cells expressing PS were determined in (**a**) (*i*) HK1 and (**b**) (*i*) NP69 upon H_2_O_2_ treatment. Means and SD from three independent experiments carried out in duplicate are shown. Student’s *t* test was used for statistical analysis to compare treated groups with untreated control, *p < 0.05. Representative *dot plot diagrams* showing the apoptotic populations of (**a**) (*ii*) H_2_O_2_-treated HK1 cells and (**b**) (*ii*) H_2_O_2_-treated NP69 cells detected by Annexin V-FITC and PI staining are shown. The* lower left* quadrants show viable cells; the* lower right* quadrants represent early apoptotic cells; the* upper right* quadrants show late apoptotic and necrotic cells

In order to determine the apoptosis-inducing effect of H_2_O_2_, the H_2_O_2_-treated HK1 cells were subjected to the analysis of phosphatidylserine (PS) externalization by flow cytometry. As shown in Fig. 1a i, treatment of HK1 cells with 50 μM of H_2_O_2_ for 4 and 8 h resulted in 1.2-fold (*p* value = 0.031) to 1.7-fold (*p* value <0.001) increase in apoptosis as compared with the untreated control. The apoptosis-inducing effect of H_2_O_2_ was also tested in NP69 cells. As shown in Fig. 1b i, the percentages of apoptosis detected in NP69 cells treated with 100 μM of H_2_O_2_ for 16 and 24 h were 2.8-fold (*p* value <0.001) to 2.9-fold (*p* value <0.001) higher than the untreated control. Majority of cells in the untreated HK1 and NP69 showed no measurable apoptosis. The low percentage of apoptosis observed in the untreated samples was due to spontaneous cell death. To serve as a positive control, camptothecin (CPT) was used to induce apoptosis in HK1 and NP69 cells. CPT is a well-known apoptotic inducer. It has been shown that NPC cells could be induced to undergo apoptosis with 2–10 µM of CPT [33]. Representative dot plot diagrams showing the apoptotic populations of H_2_O_2_-treated HK1 and NP69 cells were shown in Fig. 1a ii and Fig. 1b ii respectively. Collectively, these findings suggest that H_2_O_2_ was able to induce apoptosis in both HK1 and NP69 cells.

### H_2_O_2_ induces mitochondrial membrane potential (MMP) disruption in HK1 and NP69 cells

**Fig. 2:**
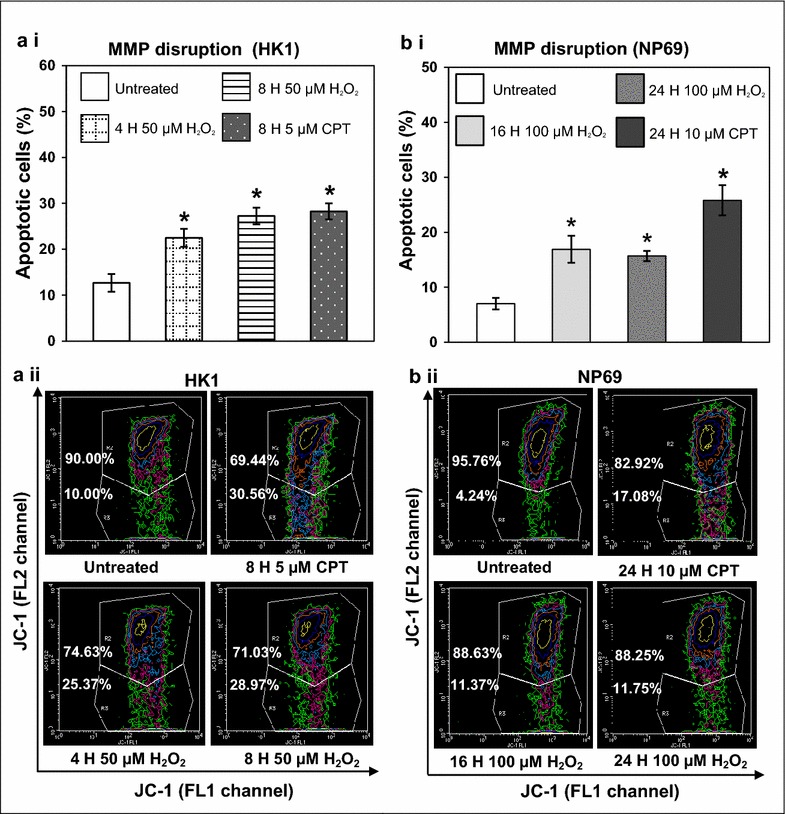
H_2_O_2_ induces MMP disruption in HK1 and NP69 cells. HK1 and NP69 cells were treated with H_2_O_2_ as described in Fig. 1. The cells were then subjected to flow cytometric analysis of MMP disruption as described in “Methods” section. Percentages of apoptotic cells showing MMP disruption were determined in (**a**) (*i*) HK1 and (**b**) (*i*) NP69 after treatment with H_2_O_2_. Means and SD of two independent experiments performed in duplicate are shown. The differences between untreated control and treated groups were compared by using Student’s *t* test, *p < 0.05. Representative* contour plot* diagrams showing the apoptotic populations of (**a**) (*ii*) H_2_O_2_-treated HK1 cells and (**b**) (*ii*) H_2_O_2_-treated NP69 cells determined by JC-1 staining are shown. The* upper* quadrants represent viable cells while the* lower* quadrants show apoptotic cells

The apoptosis-inducing effect of H_2_O_2_ was also determined by mitochondrial membrane potential (MMP) analysis using flow cytometry. H_2_O_2_-treated HK1 (Fig. 2a i) and NP69 (Fig. 2b i) cells show a significant loss of MMP. Majority of cells in the untreated HK1 and NP69 did not show sign of apoptosis. CPT was used for apoptosis induction in HK1 and NP69 cells to serve as a positive control. The percentages of cell showing MMP disruption were 1.8-fold (*p* value <0.001) to 2.1-fold (*p* value <0.001) higher in HK1 cells treated with 50 μM of H_2_O_2_ for 4 and 8 h as compared with the untreated control (Fig. 2a i). An approximately 2.4-fold (*p* value <0.001) and 2.2-fold (*p* value <0.001) increase in loss of MMP was observed in NP69 cells treated with 100 μM of H_2_O_2_ for 16 and 24 h respectively (Fig. 2b i). Representative contour plot diagrams showing the apoptotic populations of H_2_O_2_-treated HK1 and NP69 cells were shown in Fig. 2a ii and Fig. 2b ii respectively. Taken together, these results strengthen the evidence that H_2_O_2_ is a potential apoptotic inducer in both HK1 and NP69 cells.

### H_2_O_2_ induces caspase activity in HK1 and NP69 cells that are inhibited by caspase inhibitor

**Fig. 3:**
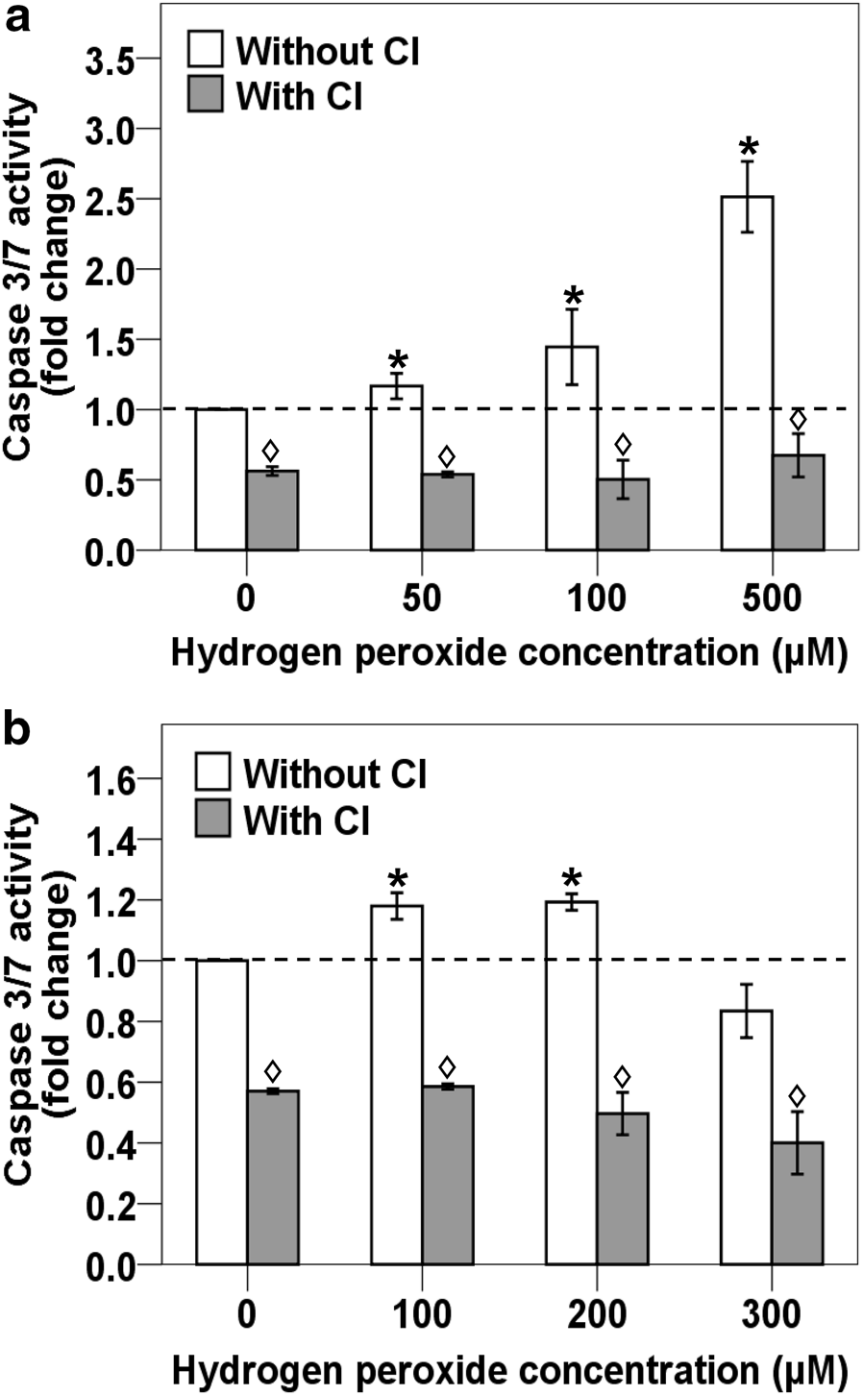
H_2_O_2_-induced apoptosis involves caspase activity that is blocked by caspase inhibitor. HK1 and NP69 cells were left untreated or pre-treated with 50 µM of Z-DEVD-FMK (Caspase-3 Inhibitor II) for 1 h. HK1 cells were then either untreated or treated with 50, 100 and 500 µM of H_2_O_2_ for 8 h, whereas NP69 cells were either untreated or treated with 100, 200 and 300 µM of H_2_O_2_ for 16 h. Activity of caspase-3/7 was measured by using Caspase-Glo 3/7 Assay in (**a**) HK1 and (**b**) NP69 cells. Means and SD of two independent experiments performed in duplicate are shown. Data are expressed as fold change normalized to untreated control. *Asterisks* indicates significant increase in H_2_O_2_-treated cells as compared with untreated control, *open diamond* indicates significant decrease in CI-pretreated cells as compared with its corresponding sample without pretreatment of CI (*P* < 0.05, Student’s *t* test)

To test if this H_2_O_2_-induced apoptosis is caspase-dependent, the activity of the main effectors, caspase-3/7, was determined using the Caspase-Glo 3/7 Assay. As shown in Fig. 3a, treatment of HK1 cells for 8 h with 50, 100 and 500 μM of H_2_O_2_ resulted in 1.2-fold (*p* = 0.015), 1.4-fold (*p* = 0.045) to 2.5-fold (*p* = 0.001) increase in caspase-3/7 activity as compared with the untreated control. The caspase-3/7 activity was also measured in H_2_O_2_-treated NP69 cells. As shown in Fig. 3b, the caspase-3/7 activity detected in NP69 cells treated for 16 h with 100 and 200 μM of H_2_O_2_ were both 1.2-fold (*p* value <0.001) higher than the untreated control. Surprisingly, the caspase-3/7 activity was decreased in NP69 cells treated with 300 μM for 16 h. In all samples of both HK1 and NP69 cells pretreated with Z-DEVD-FMK (Caspase-3 Inhibitor II), the caspase-3/7 activity was inhibited (*p* value <0.001).

### H_2_O_2_ induces *AF9* cleavages in HK1 cells that are abolished by caspase inhibitor

**Fig. 4:**
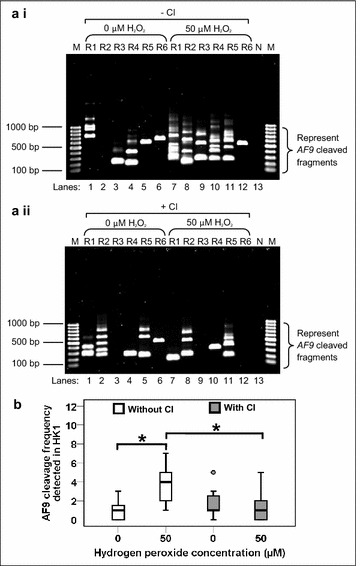
H_2_O_2_ induces *AF9* cleavages in HK1 cells that are abolished by caspase inhibitor. (**a**) Representative gel pictures showing the IPCR analysis of H_2_O_2_-treated HK1 cells: (*i*) without CI pre-treatment (*ii*) with CI pre-treatment. HK1 cells were left untreated or pre-treated with 50 µM of Z-DEVD-FMK (Caspase-3 Inhibitor II) for 1 h. The cells were then either untreated (*Lanes 1*–*6*) or treated with 50 µM of H_2_O_2_ for 8 h (*Lanes 7*–*12*). Genomic DNA was extracted and modified for nested IPCR as described in “Methods” section. Six replicates (R1–R6) were prepared for each cell sample in nested IPCR. The IPCR bands derived from the *AF9* cleaved chromosome were indicated by the side brackets. N: Negative control for IPCR. M: 100 bp DNA ladder. (**b**) *AF9* cleavage frequency detected in HK1 cells. Data are representative of two independent experiments. Each experiment consisted of 6 IPCR replicates for each cell sample. The results are presented as medians with IQRs. *P < 0.05 (Mann–Whitney *U* test)

Our next step was to investigate if oxidative stress-induced apoptosis does lead to cleavage of the *AF9* gene. Upon H_2_O_2_ treatment, genomic DNA extraction was performed in HK1 cells for nested Inverse Polymerase Chain Reaction (IPCR). IPCR primers were designed to identify chromosomal breaks within the breakpoint cluster region, BCR1 (intron 4 telomeric end) of the *AF9* gene [34]. Based on the primers positions, the IPCR product will be approximately 950 bp if there is no breakage within the *AF9* gene. If there is any breakage within the *AF9* gene, IPCR products of less than 950 bp should be obtained. As shown in Fig. 4a i, H_2_O_2_-treated HK1 cells show numerous IPCR bands of less than 950 bp which represent the cleaved *AF9* gene (Lanes 7–12). A few IPCR bands were also detected in the untreated HK1 cells (Lanes 1, 3, 4, 5 and 6). As discussed in the flow cytometric analyses, untreated sample contained a small amount of unhealthy cells. These dying cells might undergo endogenous DNA breaks and contribute to the background. As shown in Fig. 4b, the median cleavage frequency of the *AF9* gene detected in H_2_O_2_-treated HK1 cells was 4.0-fold higher than that of untreated HK1 cells (*p* = 0.001). These findings clearly demonstrate that oxidative stress-induced apoptosis in HK1 cells leads to cleavages within the *AF9* gene.

In order to investigate the role of CAD in chromosomal breaks mediated by oxidative stress-induced apoptosis, caspase inhibition assay was conducted. When an apoptotic stimulus such as H_2_O_2_ is present, caspase-3 will be activated. The ICAD which exists naturally as a complex with CAD will be cleaved by caspase-3. Subsequently, CAD is released from ICAD and degrades the chromosomal DNA [29, 30]. Therefore, if CAD does play a role in mediating chromosomal breaks in H_2_O_2_-induced apoptosis, by inhibiting caspase-3, we expect to see reduction or elimination of chromosomal breaks in H_2_O_2_-treated cells. From the IPCR result obtained from the caspase inhibition assay (Fig. 4a ii), it was obvious that the chromosomal breaks in H_2_O_2_-treated HK1 cells have been reduced (Lanes 7–12). The *box plot* in Fig. 4b reveals that, by inhibiting caspase-3, H_2_O_2_-treated HK1 cells showed a 4.0-fold reduction in the median cleavage frequency of the *AF9* gene (*p* = 0.005).

### H_2_O_2_ induces *AF9* cleavages in NP69 cells that are abolished by caspase inhibitor

**Fig. 5:**
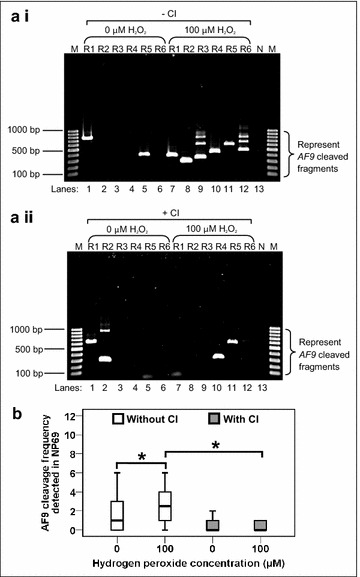
H_2_O_2_ induces *AF9* cleavages in NP69 cells that are abolished by caspase inhibitor. (**a**) Representative gel pictures showing the IPCR analysis of H_2_O_2_-treated NP69 cells: (*i*) without CI pre-treatment (*ii*) with CI pre-treatment. NP69 cells were left untreated or pre-treated with 50 µM of Z-DEVD-FMK (Caspase-3 Inhibitor II) for 1 h. The cells were then either untreated (*Lanes 1*–*6*) or treated with 100 µM of H_2_O_2_ for 16 h (*Lanes 7*–*12*). Genomic DNA was extracted and modified for nested IPCR as described in “Methods” section. Six replicates (R1–R6) were prepared for each cell sample in nested IPCR. The IPCR bands derived from the *AF9* cleaved chromosome were indicated by the* side brackets*. N: Negative control for IPCR. M: 100 bp DNA ladder. (**b**) *AF9* cleavage frequency detected in NP69 cells. Data are representative of two independent experiments. Each experiment consisted of 6–8 IPCR replicates for each cell sample. The results are expressed as medians with IQRs. *P < 0.05 (Mann–Whitney *U* test)

To confirm that the effect of H_2_O_2_ was not cell line-specific, H_2_O_2_ treatment was also carried out in NP69 cells. Figure 5a i shows the representative IPCR result of NP69 cells after treatment with H_2_O_2_. Two IPCR bands were detected in the untreated sample (Lanes 1 and 5) which might be due to endogenous DNA breaks. In contrast, there were numerous IPCR bands observed in the H_2_O_2_-treated sample (Lanes 7–12). The median cleavage frequency of the *AF9* gene detected in H_2_O_2_-treated NP69 cells was 2.5-fold higher than that of untreated NP69 cells (*p* = 0.038) (Fig. 5b). Our findings clearly demonstrate that oxidative stress-induced apoptosis in NP69 cells leads to *AF9* gene cleavages.

To further strengthen our observation that H_2_O_2_-induced chromosomal breaks were mediated by CAD, caspase inhibition assay was also performed in NP69 cells. As shown in Fig. 5a ii, in the presence of caspase inhibitor, the chromosomal breaks in H_2_O_2_-treated NP69 cells were obviously reduced (Lanes 7–12). Upon caspase inhibition, the median cleavage frequency of the *AF9* gene in H_2_O_2_-treated NP69 cells has been decreased from 2.5 to zero (*p* value <0.001) (Fig. 5b). In summary, our findings clearly establish that CAD plays an important role in mediating chromosomal breaks in H_2_O_2_-induced apoptosis.

### H_2_O_2_ induces cleavages within the *AF9* region that translocates with the mixed lineage leukemia (*MLL)* gene

**Fig. 6:**
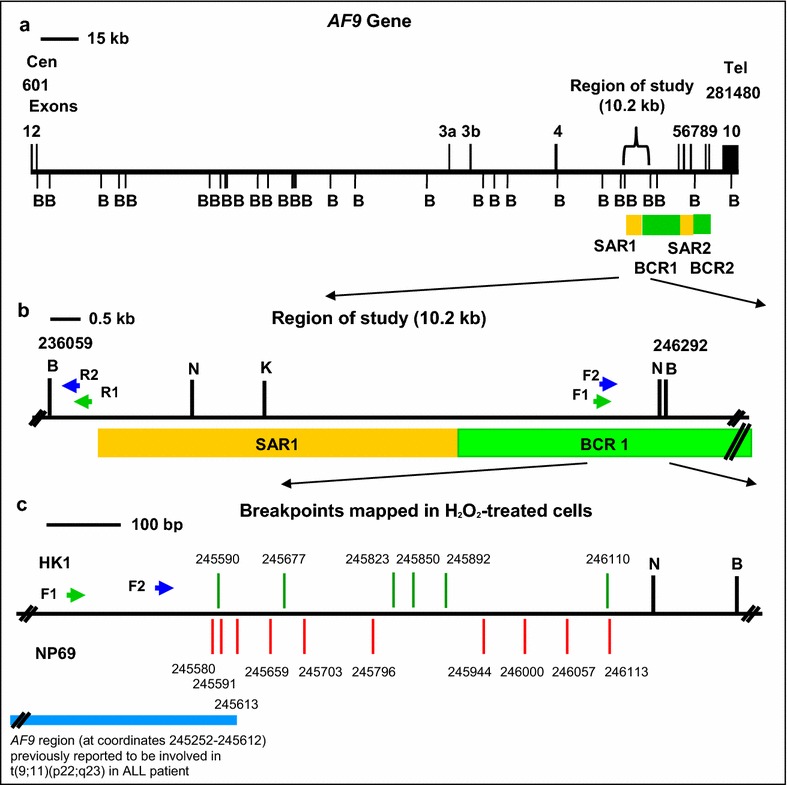
A map illustrating the positions of H_2_O_2_-induced chromosomal breaks within the *AF9* gene. (**a**) The *AF9* genomic map from nucleotide positions 601-281480 [EMBL:ENSG00000171843]. *Black vertical lines* show the locations of exons 1–10. *Green boxes* represent the two previously identified patient breakpoints cluster regions which are indicated as BCR1 and BCR2 [34]. *Yellow boxes* show the two previously experimentally isolated MAR/SAR which are indicated as SAR1 and SAR2 [34]. *Bam*H I (B) restriction sites are shown. **b** The region of study (10.2 kb). *Bam*H I (B), *Kpn* I (K) and *Nde* I (N) restriction sites are shown.* Green arrows* represent the primers (R1, AF9 236451 R and F1, AF9 245385 F) used in the first round of nested IPCR while* blue arrows* represent the primers (R2, AF9 236211 R and F2, AF9 245507 F) used in the second round of nested IPCR. (**c**) Breakpoints mapped in H_2_O_2_-treated cells. *Green and red vertical lines* represent the presently detected breakpoints in H_2_O_2_-treated HK1 and NP69 cells respectively. All the chromosomal breaks were mapped within BCR1 in close proximity with SAR1. *Blue box* represents the *AF9* region (at coordinates 245252-245612) that translocates with the *MLL* gene, resulted in *MLL*-*AF9* fusion gene previously identified in ALL patient [GenBank:AM050804]

Some of the IPCR bands were excised, purified and sequenced in order to confirm that these fragments were derived from the cleaved *AF9* gene. The sequencing data reveal that they are all derived from the cleaved *AF9* gene. The breakpoints identified in H_2_O_2_-treated HK1 and NP69 cells are shown in Table 1. Interestingly, a few breakpoints (at coordinates 245,580, 245,590, 245,591) are mapped within the region of *AF9* (at coordinates 245,252–245,612) that was previously reported to be involved in t(9;11)(p22;q23). This translocation resulted in mixed lineage leukemia (*MLL*)-*AF9* fusion gene, previously identified in ALL patient [GenBank:AM050804]. A breakpoint similar with that identified in ALL patient is mapped at coordinate 245,613 [GenBank:AM050804]. A few breakpoints mapped at coordinates 245,590, 245,591, 246,110 and 246,113, are similar with those reported in cultured normal blood cells and CEM cells treated with etoposide (VP16) [35]. A map representing the positions of H_2_O_2_-induced chromosomal breaks in HK1 and NP69 cells within the *AF9* gene is illustrated in Fig. 6.

**Table 1:** Breakpoints detected within the *AF9* gene in H_2_O_2_-treated cells

Cell line treated with H_2_O_2_	Breakpoint	Remarks
HK1	245,590	This chromosomal break falls within the *AF9* region (at coordinates 245252–245612) that translocates with the *MLL* gene, resulted in *MLL*-*AF9* fusion gene previously identified in ALL patient [GenBank:AM050804]. This breakpoint is three nucleotides different from a breakpoint (at coordinate 245593) reported in cultured normal blood cells treated with VP16 [35]
	245,677	
	245,823	
	245,850	
	245,892	
	246,110	This breakpoint is four nucleotides different from a breakpoint (at coordinate 246114) reported in CEM cells treated with VP16 [35]
NP69	245,580	This chromosomal break falls within the *AF9* region (at coordinates 245252-245612) that translocates with the *MLL* gene, resulted in *MLL*-*AF9* fusion gene previously identified in ALL patient [GenBank:AM050804]
	245,591	This chromosomal break falls within the *AF9* region (at coordinates 245252-245612) that translocates with the *MLL* gene, resulted in *MLL*-*AF9* fusion gene previously identified in ALL patient [GenBank:AM050804]. This breakpoint is two nucleotides different from a breakpoint (at coordinate 245593) reported in cultured normal blood cells treated with VP16 [35]
	245,613	This breakpoint is 1 nucleotide different from a breakpoint (at coordinate 245612) identified in ALL patient [GenBank:AM050804]
	245,659	
	245,703	
	245,796	
	245,944	
	246,000	
	246,057	
	246,113	This breakpoint is one nucleotide different from a breakpoint (at coordinate 246114) reported in CEM cells treated with VP16 [35]

The nucleotide positions of the chromosomal breaks detected within the *AF9* gene were mapped according to the *AF9* sequence accessed from Ensembl database [EMBL:ENSG00000171843]

## Discussion

Chromosomal rearrangements such as deletion are commonly found in NPC [36]. However, the mechanism of rearrangement is unknown. In addition, although oxidative stress has well been associated with carcinogenesis, the underlying mechanism is not well understood. In view that apoptosis has been implicated in chromosomal translocation in leukemia [11], we intend to examine if the apoptotic process plays a role in chromosomal rearrangement in NPC, especially during oxidative stress.

The *AF9* gene located at 9p22 was targeted in this study. This gene was chosen because it locates at a common chromosomal deletion site in NPC [32]. Besides, the reciprocal translocation of *AF9* gene with the *MLL* gene has been strongly implicated in acute myelogenous leukemia (AML), less often in ALL, in myelodysplastic syndromes (MDS) and in therapy-related AML (t-AML) [34, 37].

Both PS externalization and loss of MMP are known to be early events of apoptosis [38, 39]. The disruption of MMP promotes PS externalization and induces DNA fragmentation during apoptosis [39]. We detected significant percentages of apoptotic cells upon H_2_O_2_ treatments in both flow cytometric analyses of PS externalization and of MMP disruption. These findings indicate that H_2_O_2_ is a potential apoptotic inducer in both HK1 and NP69 cells.

Our results show that higher concentration and longer durations of H_2_O_2_ were required to induce apoptosis in normal nasopharyngeal epithelial cells (NP69) compared with NPC cells (HK1). This could be due to the reason that genomic instability is higher in cancer cells than normal cells [40, 41]. Genomic instability refers to an elevated propensity of alteration in the genome during the life cycle of cells [41]. The genome of cancer cells is commonly much more complex and unstable. Intrachromosomal instability in cancer cells implies an increased rate of occurrence of mutations. This may arise from the elevated rate of DNA damage defeating the capability of normal DNA repair systems to restore the genomic integrity. Other possible factor is that cancer cells possess defective DNA repair system which is unable to overcome normal rate of DNA damage generated through normal cellular and environmental mechanisms [40]. Apoptosis is usually triggered to control cell fate in response to extensive DNA damage. Apoptosis plays an essential role as a prominent route of cell inactivation to eliminate severely damaged cells from the dividing pool [41, 42]. Treatment with H_2_O_2_ induces caspase activation which subsequently leads to DNA fragmentation and apoptosis [43, 44]. H_2_O_2_ also interacts with cellular molecules such as DNA and cause oxidative DNA damage [45]. Upon H_2_O_2_ treatment, cells tried to evade from apoptosis by repairing the DNA lesions caused by apoptotic DNA fragmentation or oxidative DNA damage. Under oxidative stress, cancer cells with higher genomic instability which implies an elevated rate of DNA damage or an impaired DNA damage response are therefore more vulnerable to apoptosis compared with normal cells.

Both caspase-3 and caspase-7 have been well recognized as effector caspases in apoptosis (reviewed in [46]). By using a luminescence based assay, Caspase-Glo 3/7, activation of caspase-3/7 was detected in H_2_O_2_-treated HK1 and NP69 cells. This activity was inhibited by Z-DEVD-FMK (Caspase-3 Inhibitor II). DEVD inhibitor has been shown to be the most potent inhibitor of caspase-3 and caspase-7 with the lowest inhibitory constant (*Ki*) against caspase-3 (*Ki* = 0.23 nM) and caspase-7 (*Ki* = 1.6 nM) [47]. Our results indicate that H_2_O_2_ induces apoptosis in HK1 and NP69 cells in a caspase-dependent manner. It is noteworthy that when higher concentration of H_2_O_2_ (300 μM) was used to treat NP69 cells for 16 h, the cells underwent another form of cell suicide, that is, necrosis which is usually accompanied by cell lysis [48]. Almost all the attached cells showed rupture of cell membranes (data not shown). This indicates that in higher concentration, H_2_O_2_ induces necrosis rather than apoptosis. These findings are similar with those of a few researchers who discovered that low concentrations of H_2_O_2_ induced cell death with apoptotic morphologic evidence while high concentrations of H_2_O_2_ induced cell death with no apoptotic evidence in osteoblast [49, 50].

IPCR was employed to detect chromosomal breaks mediated by oxidative stress-induced apoptosis in HK1 and NP69 cells. In both cell lines, we detected a significant higher gene cleavage frequency in the H_2_O_2_-exposed cells as compared with the untreated control. We observed that both HK1 and NP69 cells have spontaneous breaks in the absence of any H_2_O_2_ treatment. These could be because cells were undergoing spontaneous cell death in culture. In summary, our results show that oxidative stress can induce apoptosis in NPC and normal nasopharyngeal epithelial cells, and cause cleavages within the *AF9* gene. This is in line with other report [51], which showed that treatment of U937 leukemic cells with H_2_O_2_ produced High Molecular Weight (HMW) DNA fragments of 50–100 kb in size. Their studies also showed that the formation of these HMW DNA fragments was mediated by topoisomerase II (TOP2) [51]. It has been demonstrated that H_2_O_2_ induced DNA fragmentation with nucleosomal intervals in caspase-3 expressing MCF-7 breast carcinoma cells which suggested that caspase-3 is a crucial player in nuclear events [52].

As discussed above, our data are consistent with the reports that H_2_O_2_ could trigger chromosomal breaks mediated by apoptosis. Following this, the nuclease acting as the key player in mediating these oxidative stress-induced chromosomal breaks was investigated. H_2_O_2_ has been found to be able to induce TOP2-mediated excision of chromosomal DNA loops which generates 50–100 kb HMW fragments as an early event in apoptosis [51]. In addition, it has been proposed that the binding and activation of CAD by TOP2 may be responsible for excision of chromosomal loops at early stage of apoptosis [53–55]. It was also demonstrated that H_2_O_2_ induced apoptotic DNA fragmentation with nucleosomal intervals in caspase-dependent manner [52]. Hence, it is possible that chromosomal breaks mediated by stress-induced apoptosis in NPC and normal nasopharyngeal epithelial cells are CAD-dependent. To explore this possibility, caspase inhibition assay was performed. In non-apoptotic cells, CAD which can be activated by caspase-3 exists naturally as a heterodimer with its inhibitor, ICAD. When an apoptotic stimulus such as H_2_O_2_ is present, caspase-3 will be activated and the ICAD which possesses two caspase-3 cleavage sites will be cleaved by caspase-3. Subsequently, CAD is released from ICAD and cleaves DNA by generating DSB [29, 30]. Therefore, if CAD does play a role in mediating chromosomal breaks in H_2_O_2_-induced apoptosis, by inhibiting caspase-3, we expect to observe reduction or elimination of chromosomal breaks in H_2_O_2_-treated cells.

DEVD cleaving caspase-3 is the primary inactivator of ICAD and therefore the primary activator of apoptotic DNA fragmentation [56]. Thus, by using Z-DEVD-FMK to inactivate caspase-3 is the most effective way of inactivating CAD. Caspase inhibition by DEVD inhibitor significantly reduced *AF9* cleavage in both H_2_O_2_-treated HK1 and NP69 cells, suggested that H_2_O_2_-induced chromosomal breaks during apoptosis involves caspase activation. These findings are consistent with those reported by Kim and colleagues (2000) where H_2_O_2_ induced DNA fragmentation in a caspase-3-dependent manner [52]. Given that activated caspase-3 can activate CAD which is responsible for DNA fragmentation, it was highly likely that H_2_O_2_-induced DNA fragmentation was CAD-dependent. Although there were some remaining breaks detected in the presence of CI, these were most probably contributed by those cells that were naturally dying in culture before addition of CI.

Chromosomal cleavages occur during DNA fragmentation in apoptosis and also during chromosomal rearrangements. It has recently become apparent that chromosomal breaks do not randomly distribute throughout a gene but usually cluster in certain regions containing specific chromatin structures such as matrix association region/scaffold attachment region (MAR/SAR) sequence [34, 57]. MAR/SAR sequences are the sites for the binding of DNA loop structure to nuclear scaffold/matrix proteins [58]. MAR/SAR sequences have been found to have DNA unwinding properties which enable them to play an important role in facilitating the entry of protein factors involved in transcription, replication, chromosome condensation and apoptosis [59, 60]. Besides, the unwinding properties also make MAR/SAR sequences to be the regions of DNA fragility which are hypersensitive to DNA breakage [60, 61]. Two patient breakpoint cluster regions (BCR) have been identified in the *AF9* gene, namely, BCR1 (within intron 4) and BCR2 (spans introns 7 and 8). The *AF9* BCRs are bordered by two MAR/SARs which were designated as SAR1 (located in intron 4) and SAR2 (spans from exons 5 to 7) [34, 57].

**Fig. 7:**
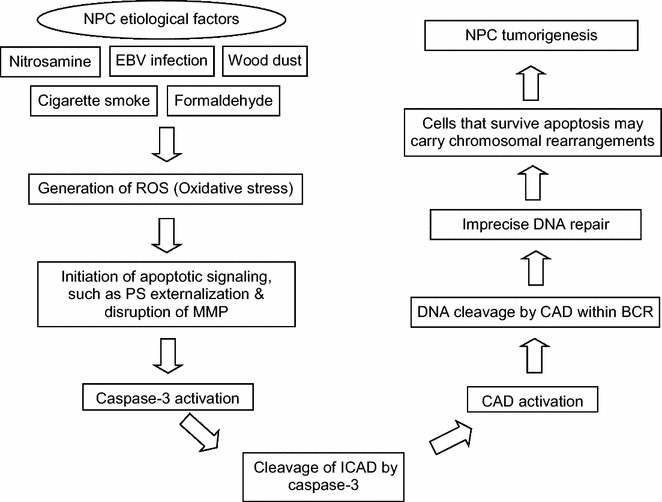
A potential model for oxidative stress-induced chromosomal rearrangement in NPC. NPC etiological factors, such as EBV infection, nitrosamine, cigarette smoke, wood dust and formaldehyde may trigger oxidative stress. PS externalization and disruption of MMP, which are recognized as apoptotic signaling, initiate oxidative stress-induced apoptosis. This in turn leads to caspase-3 activation. Upon cleavage of ICAD by caspase-3, CAD is released from its chaperone, ICAD, to cleave chromosomal DNA within BCR. The cells try to evade from apoptosis through imprecise DNA repair. Cells that survive apoptosis may carry chromosomal rearrangements such as deletion and amplification which contribute to tumorigenesis of NPC

Our findings demonstrate that oxidative stress-induced apoptosis leads to cleavages within the *AF9* BCR1 which is bordered by SAR1 and SAR2 (Fig. 6). The *AF9* BCRs have been found to share similar structural elements as the *MLL* BCR. Both *AF9* BCR*s* and *MLL* BCR were found to contain MAR/SAR sequences, topo II cleavage sites and DNase I hypersensitive (HS) cleavage sites. These structural similarities could act as recombination hot spots leading to *MLL*/*AF9* translocations in leukemia [34]. This is further supported by our result that a few chromosomal breaks were mapped within the region of *AF9* that is involved in the formation of *MLL*-*AF9* fusion gene, previously identified in ALL patient [GenBank:AM050804]. Previous studies have revealed that MAR/SAR may be a preferential site of chromosomal breaks, and may play an important role in apoptosis [62] and chromosomal rearrangements [57, 58]. During the initial stage of apoptosis, DNA fragmentation happens. Chromosome breaks when the DNA loop structure is being cleaved at the base of the DNA loop which attaches to the nuclear matrix or scaffold via MAR/SAR sequence [63, 64]. Interestingly, CAD has been found to associate with the nuclear matrix of cells undergoing apoptosis [62]. In addition, MAR/SAR sequences have also been implicated in illegitimate recombination [58]. Based on the literature and our current results, a potential model for oxidative stress-induced chromosomal rearrangements in NPC is proposed (Fig. 7).

## Conclusions

Our findings clearly demonstrate that oxidative stress-induced apoptosis may cause chromosomal cleavages in both normal epithelial nasopharyngeal and NPC cells. Therefore, oxidative stress-induced apoptosis could be one of the mechanisms underlying the chromosomal rearrangements in NPC. Besides, apoptotic nuclease CAD could be an important player in chromosomal cleavages mediated by oxidative stress-induced apoptosis.

Since most of the etiological factors of NPC are known to induce oxidative stress, antioxidants could be a preventive care for NPC. Oxidative stress has not only been implicated in NPC, but also in many other types of cancers. Therefore, the potential model that we propose for oxidative stress-induced chromosomal rearrangements in NPC may also be applicable for other types of cancers.

## Methods

### Cell lines and chemicals

HK1 NPC cell line and NP69 normal nasopharyngeal epithelial cell line were kind gifts from Prof. Tsao Sai Wah (The University of Hong Kong, Hong Kong, China) and Prof. Lo Kwok Wai (The Chinese University of Hong Kong, Hong Kong, China). RPMI 1640 and Keratinocyte-SFM medium, fetal bovine serum, l-glutamine, penicillin, streptomycin and StemPro ACCUTASE Cell Dissociation Reagent were procured from GIBCO, Invitrogen, USA. Hydrogen peroxide (H_2_O_2_) was purchased from MP Biomedicals, USA. Camptothecin (CPT) was bought from Santa Cruz Biotechnology, California, USA. Annexin V-Fluorescein isothiocyanate (FITC) Apoptosis Detection Kit I was obtained from BD Pharmingen™, Becton–Dickinson Biosciences, USA. Flow Cytometry Mitochondrial Membrane Potential Detection Kit was purchased from BD™ MitoScreen, Becton–Dickinson Biosciences, USA. Caspase-3 inhibitor II (Z-DEVD-FMK) was from Calbiochem, USA. Caspase-Glo 3/7 Assay Kit was bought from Promega, USA. Phenol and Sodium dodecyl sulfate (SDS) were obtained from Amresco, USA. Chloroform was purchased from R&M Chemicals, UK. Isoamylalchohol was bought from Fluka, Switzerland. Ammonium acetate was from Merck, Germany. DNA Polymerase I, Large (Klenow) Fragment, T4 DNA Ligase and all the restriction enzymes were purchased from New England Biolabs (NEB), USA. PCR primers were obtained from First Base Laboratories. dNTP mix was from Promega, USA. Phusion High-Fidelity DNA Polymerase was bought from Finnzymes, Finland. QIAquick Nucleotide Removal Kit and QIAquick Gel Extraction Kit were purchased from QIAGEN, Germany.

### Cell cultures

HK1 cells were grown in RPMI 1640 medium supplemented with 10 % (v/v) heat-inactivated fetal bovine serum, 2 mM l-glutamine, 100 U/ml penicillin and 100 µg/ml streptomycin. NP69 cells were cultured in Keratinocyte-SFM medium supplemented with 4–5 ng/ml recombinant epidermal growth factor (rEGF), 40–50 µg/ml bovine pituitary extract (BPE), 2 % (v/v) heat-inactivated fetal bovine serum, 100 U/ml penicillin and 100 µg/ml streptomycin. Cells were maintained at 37 °C with 5 % CO_2_.

### Flow cytometric analysis of phosphatidylserine (PS) externalization

HK1 cells were seeded at a density of 5.5 × 10^5^ in 150 mm culture dishes. At cell confluency of 60–70 %, the cells were either left untreated or treated with 50 µM of H_2_O_2_ for 4 and 8 h. HK1 cells treated with 5 µM of CPT for 8 h were included as a positive control. NP69 cells were plated at a density of 1.5 × 10^5^ in 150 mm culture dishes. At cell confluency of 30–40 %, the cells were either left untreated or treated with 100 µM of H_2_O_2_ for 16 and 24 h. NP69 cells treated with 10 µM of CPT for 24 h were used to serve as a positive control. The cells were then collected by using StemPro ACCUTASE Cell Dissociation Reagent. The collected cells were subjected to flow cytometric analysis of PS externalization using Annexin V-FITC Apoptosis Detection Kit I according to the manufacturer’s protocols. Briefly, the cells were suspended in Binding Buffer at a concentration of 1 × 10^6^ cells/ml. The cells were stained with Annexin V-FITC and Propidium Iodide (PI) for 15 min. The cells were then analyzed by a flow cytometer (FACSCalibur, Becton–Dickinson, USA) with a minimum of 10,000 events per sample. Cells usually expose PS when they are in early stage of apoptosis [38]. Annexin V has high affinity for PS and may be conjugated to fluorochrome such as FITC. Thus, Annexin V-FITC staining was used to detect cells undergoing early apoptosis with exposed PS. This staining was performed simultaneously with PI staining. PI can easily penetrate damaged cell membrane and subsequently intercalate into double-stranded nucleic acid. Hence, PI staining was used to detect cells which were in early apoptosis, late apoptosis, necrosis or already dead. Cells that were viable were both Annexin V-FITC and PI negative; cells that were in early apoptosis were Annexin V-FITC positive and PI negative; and cells that were in late apoptosis or necrosis were both Annexin V-FITC and PI positive. This assay was performed in duplicate.

### Flow cytometric analysis of mitochondrial membrane potential (MMP) disruption

HK1 and NP69 cells were treated as described above. The harvested cells were subjected to flow cytometric analysis of MMP disruption using Flow Cytometry Mitochondrial Membrane Potential Detection Kit according to the manufacturer’s protocols. Briefly, the collected cells were stained with JC-1 (1st J-aggregate-forming cationic dye) Working Solution for 15 min at 37 °C in a CO_2_ incubator. The cells were washed twice and then resuspended in Assay Buffer. The cells were then analyzed by a flow cytometer (FACSCalibur, Becton–Dickinson). In each cell suspension, acquisition was terminated when 10,000 events were analyzed. JC-1 is a membrane-permeable lipophilic cationic fluorochrome that is used to evaluate the status of the MMP (Δψ). JC-1 exists in two different states, aggregates or monomers which possess different emission spectrum. Both JC-1 aggregates and monomers emit fluorescence in the green end of the spectrum which is measured in the FL-1 channel of flow cytometer. In viable cells, the Δψ of healthy mitochondria is polarized. JC-1 monomers are rapidly taken up by such mitochondria leading to the formation of JC-1 aggregates within the mitochondria. JC-1 aggregates emit maximally at 590 nm and show a red spectral shift resulting in higher levels of red fluorescence emission which is detected in the FL-2 channel. When cells are undergoing apoptosis, the Δψ of unhealthy mitochondria is depolarized. JC-1 is not taken up by mitochondria with depolarized Δψ but remained in the cytoplasm as monomers. JC-1 monomers emit maximally at 527 nm which can be detected in FL-1 channel. These monomers do not have a red spectral shift and hence have lower fluorescence in the FL-2 channel. This analysis was carried out in duplicate.

### Measurement of caspase-3/7 activity

HK1 cells were seeded in 96-well plates at a density of 5 × 10^3^ per well, while NP69 cells were plated in 96-well plate at a density of 2 × 10^3^ per well. Each well contained a total volume of 100 μl of cultured medium. After 24 h, HK1 and NP69 cells were either left untreated or pre-treated with 50 μM of Z-DEVD-FMK (Caspase-3 Inhibitor II) for 1 h. Subsequently, the HK1 cells were either left untreated or co-treated with 50, 100 and 500 μM of H_2_O_2_ for 8 h, while the NP69 cells were either left untreated or co-treated with 100, 200 and 300 μM of H_2_O_2_ for 16 h. After exposure, caspase-3/7 activity was determined by using Caspase-Glo 3/7 Assay Kit (Promega) as recommended by the manufacturer. In brief, 100 μl of Caspase-Glo 3/7 reagent was added to each well of 96-well plate containing untreated control and treated cells. After incubation at room temperature for 30 min, luminescence was measured in a microplate reader (Tecan Infinte 200 Pro, Austria). The values from the treated cells were compared with the values generated from the untreated control cells to obtain the fold change. Each sample was measured in duplicate.

### Identification of oxidative stress-induced chromosomal breaks by nested IPCR

#### Apoptosis induction assay

HK1 cells were seeded in 60 mm culture dishes with a density of 8 × 10^4^. When the HK1 cells reached a confluency of 60–70 %, the cells were left untreated or treated with 50 µM of H_2_O_2_ for 8 h. NP69 cells were plated in 60 mm culture dishes with a density of 2 × 10^4^. When the NP69 cells reached a confluency of 30–40 %, the cells were treated with or without 100 µM of H_2_O_2_ for 16 h.

#### Genomic DNA extraction

Genomic DNA was extracted using phenol/chloroform/isoamyl alcohol extraction method [65] with minor modifications. Briefly, the cells were lyzed in cell lysis buffer containing 0.5 % sodium dodecyl sulfate (SDS) and 100 µg/ml proteinase K at 55 °C for 16 h. After phenol extractions, the DNA was precipitated with 0.2 volume of 7.5 M ammonium acetate and 2 volumes of absolute ethanol. The DNA was then washed with 70 % ethanol, briefly air-dried and redissolved in TE buffer, pH 8.0.

#### Manipulation of genomic DNA for nested IPCR

**Fig. 8:**
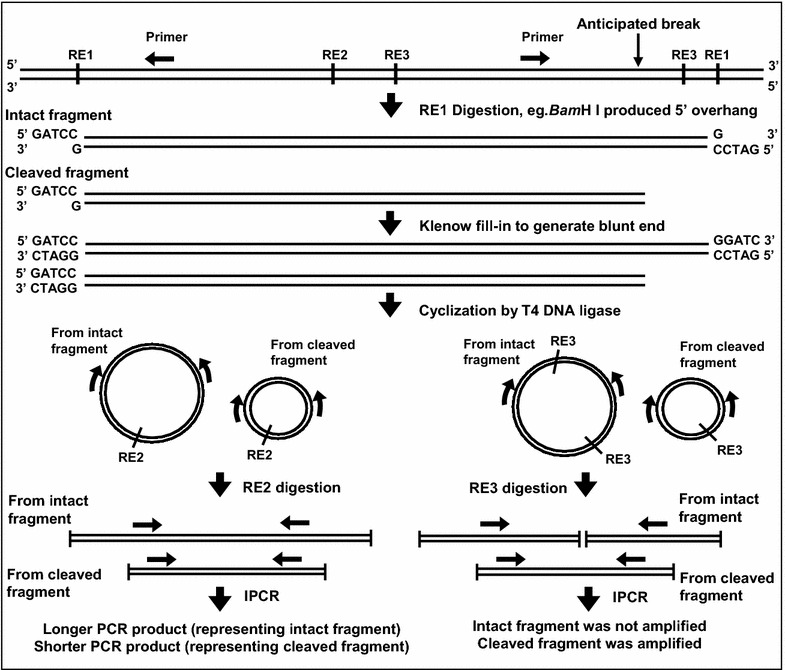
Flow chart showing the DNA manipulation steps for nested IPCR

The extracted genomic DNA was manipulated in preparation for nested IPCR. These manipulation steps were simplified in Fig. 8. The extracted genomic DNA was digested with 100 U of *Bam*H I (RE1 in Fig. 8) at 37 °C for 16 h. *Bam*H I digestion produced staggered 4 base pairs (GATC) 5′ overhang, whereas apoptotic nucleases such as CAD mainly generates blunt ends [66]. At this stage, for the intact targeted DNA fragment, both ends were *Bam*H I sites with staggered overhangs, while for the cleaved targeted DNA fragment, one end was the staggered overhang produced by *Bam*H I and the other end was the blunt end created by the apoptotic nuclease. Thus, the digested DNA was subjected to Klenow fill-into produce blunt-ended fragments. Two µg of DNA was used as template for Klenow fill-in with 2 units of DNA Polymerase I Large (Klenow) Fragment, supplemented with 33 µM of dNTP mix at 25 °C for 15 min. Cyclization was then performed at 16 °C for 16 h with 2000 units of T4 DNA ligase. Ethanol precipitation was performed with 1 volume of 3 M sodium acetate (NaAc), pH 5.2 and 2.5 volumes of ice cold absolute ethanol. The DNA pellet was washed with 70 % ethanol and air-dried, then dissolved in TE buffer, pH 8.0. The dissolved DNA was divided into two, one digested with 100 U of *Kpn* I (RE2 in Fig. 8), the other digested with 100 U of *Nde* I (RE3 in Fig. 8). These digestions were performed at 37 °C for 16 h. *Kpn* I was used to linearize the cyclized fragments. *Nde* I was used to linearize the cyclized fragments and simultaneously to eliminate the amplification of the intact fragments, thus only the cleaved fragments will be amplified. The digested DNA was purified using QIAGEN QIAquick Nucleotide Removal Kit according to the manufacturer’s protocol.

#### Nested IPCR

Nested IPCR was performed with 200 ng of DNA template, 1X of HF buffer (containing 1.5 mM of MgCl_2_), 200 µM of dNTP mix, 0.5 µM of each reverse primer and forward primer, and 0.4 U of Phusion High-Fidelity DNA Polymerase. Sterile ultrapure water in place of DNA template was included as a negative control. Cycle condition used in the first round was: 30 s of 98 °C for 1 cycle (initial denaturation), followed by 30 cycles of 98 °C for 10 s (denaturation), 69 °C for 30 s (annealing), 72 °C for 15 s (extension), followed by 1 cycle of 72 °C for 10 min (final extension). Two µl of fivefold diluted first round IPCR product was used for second round with similar cycle condition, except that the annealing temperature was 57 °C. The primers used during the first round of IPCR were 5′-ATTCTAGACCCCAAAAAATTCTCAG-3′ (reverse) and 5′-CTCTTAATGCCACTGCCATGA-3′ (forward), whereas the primers used in the second round were 5′-CATATCCTTTTCATACCTGG-3′ (reverse) and 5′-ATTGGTGTCAATCAAATGC-3′ (forward). All the IPCR amplifications were performed in a Veriti 96 Well Thermal Cycler (Applied Biosystems, USA).

### Caspase inhibition assay

HK1 cells were seeded in 60 mm culture dishes with a density of 8 × 10^4^ and grown until the cells reached a confluency of 60–70 %. NP69 cells were plated in 60 mm culture dishes with a density of 2 × 10^4^ and cultured until the cells reached a confluency of 30–40 %. HK1 and NP69 cells were either left untreated or pre-treated with 50 μM of Z-DEVD-FMK (Caspase-3 Inhibitor II) for 1 h. Subsequently, the HK1 cells were either left untreated or co-treated with 50 μM of H_2_O_2_ for 8 h, while the NP69 cells were either left untreated or co-treated with 100 μM of H_2_O_2_ for 16 h. Following that, the cells were subjected to genomic DNA extraction and IPCR detection of the chromosomal breaks as described above.

### Agarose gel electrophoresis and DNA sequencing of the cleavage bands

To detect cleaved chromosome, the IPCR products were analyzed on 1 % agarose gel and stained with ethidium bromide. The IPCR bands representing the *AF9* cleaved fragments were excised, purified with QIAGEN QIAquick Gel Extraction Kit and sequenced. The sequencing data obtained was annotated by blasting the human genome database (Genomic BLAST, http://www.ncbi.nlm.nih.gov/genome/seq/Blast). By using Seqman DNASTAR software (Lasergene, USA), the sequencing data was analyzed and aligned with the published *AF9* gene sequence [EMBL:ENSG00000171843] to determine the breakpoints of the cleaved fragments. A genomic map illustrating the locations of the detected chromosomal breaks was constructed.

### Quantification of gene cleavage frequency

The nested IPCR amplifications were performed in 6–8 replicates per cell sample in each experiment. The number of IPCR bands representing the *AF9* cleaved fragments was counted. Gene cleavage frequency represents the median number of *AF9* cleaved fragments identified in two independent experiments.

### Statistical analysis

All experiments were repeated at least once independently showing similar results. The Student’s *t* test was used to compare the untreated control and treated groups in flow cytometric analyses and measurement of caspase activity. Data for flow cytometric analyses and measurement of caspase activity are presented as mean with standard deviation (SD). The Mann–Whitney *U* test was used to evaluate the significance of differences in the gene cleavage frequency detected by nested IPCR. Data for IPCR are reported as median and interquartile range (IQR). All statistical tests are two sided. Differences were considered statistically significant at *p* value <0.05.

## Abbreviations

NPC: nasopharyngeal carcinoma
CAD: caspase-activated deoxyribonuclease
ROS: reactive oxygen species
H_2_O_2_: hydrogen peroxide
IPCR: inverse polymerase chain reaction
ALL: acute lymphoblastic leukemia
EBV: Epstein-Barr virus
DSB: DNA double-strand breaks
NHEJ: non-homologous end joining
ICAD: inhibitor of caspase-activated deoxyribonuclease
PS: phosphatidylserine
CPT: camptothecin
MMP: mitochondrial membrane potential (Δψ)
BCR: breakpoint cluster region
VP16: etoposide
HMW: high molecular weight
TOP2: topoisomerase II
MAR/SAR: matrix association region/scaffold attachment region
PI: propidium iodide
CI: caspase inhibitor
